# Supplementary material for: Empirical methods for controlling false positives and estimating confidence in ChIP-Seq peaks
Source: BMC Bioinformatics. 2008 Dec 5;9:523. doi: 10.1186/1471-2105-9-523 (PMC2628906; doi:10.1186/1471-2105-9-523)
Supplement: Additional File 1 — A variety of html documents from the USeq web site detailing the available applications, their best usage, output file type descriptions, command line menus, etc. [file 1471-2105-9-523-S1.zip › USeqUserGuides/JavaDoc.zip!/JavaDoc/util/bio/parsers/gff/ExtractGffFeatures.html]

ExtractGffFeatures


|  |  |  |
| --- | --- | --- |
| **Overview** \| **Package** \| **Class** \| **Use** \| **Tree** \| **Deprecated** \| **Index** \| **Help** | |  |
| **PREV CLASS**   **NEXT CLASS** | **FRAMES**    **NO FRAMES**     **All Classes** |  |
| SUMMARY: NESTED \| FIELD \| CONSTR \| METHOD | DETAIL: FIELD \| CONSTR \| METHOD |  |


---


## util.bio.parsers.gff Class ExtractGffFeatures

```
java.lang.Object
  util.bio.parsers.gff.ExtractGffFeatures
```

---

``` public class ExtractGffFeatures extends java.lang.Object ```

---

**Constructor Summary**

`ExtractGffFeatures(java.lang.String[] args)`


| **Method Summary** | |
| --- | --- |
| `void` | `extractGffFeatures()` |
| `static void` | `main(java.lang.String[] args)` |
| `void` | `printClusters(java.util.LinkedHashMap map)`             Prints all the gff lines. |
| `static void` | `printDocs()` |
| `void` | `printUCSCFormat(java.util.LinkedHashMap map)`             Print each cluster in ucsc table format refGene.name refGene.chrom refGene.strand refGene.txStart refGene.txEnd refGene.cdsStart refGene.cdsEnd refGene.exonCount refGene.exonStarts refGene.exonEnds refLink.product geneName.name refSeqSummary.summary NM\_198576 chr1 + 995569 1031415 995619 1030284 36 995569,997647,1010723,1016111,1016522,1016827,1017258,1018541,1018840,1019125,1019411,1019636,1020463,1020661,1021035,1021266,1021462,1021699,1022122,1022629,1022875,1023078,1023314,1024169,1024538,1024868,1025205,1025535,1025729,1026028,1026555,1026755,1027030,1029055,1029750,1030126, 995820,997909,1010771,1016327,1016747,1017052,1017465,1018760,1019035,1019326,1019560,1019742,1020580,1020826,1021179,1021391,1021568,1022038,1022260,1022757,1022990,1023198,1023668,1024362,1024754,1025098,1025340,1025632,1025894,1026140,1026672,1026948,1027118,1029280,1029854,1031415, agrin AGRIN Agrin is a neuronal aggregating factor that induces the aggregation of ... |
| `void` | `processArgs(java.lang.String[] args)`             This method will process each argument and assign new varibles |

**Methods inherited from class java.lang.Object**

`equals, getClass, hashCode, notify, notifyAll, toString, wait, wait, wait`

**Constructor Detail**

### ExtractGffFeatures

```
public ExtractGffFeatures(java.lang.String[] args)
```


**Method Detail**

### extractGffFeatures

```
public void extractGffFeatures()
```

---


### printClusters

```
public void printClusters(java.util.LinkedHashMap map)
```

:   Prints all the gff lines.

---


### printUCSCFormat

```
public void printUCSCFormat(java.util.LinkedHashMap map)
```

:   Print each cluster in ucsc table format
    refGene.name refGene.chrom refGene.strand refGene.txStart refGene.txEnd refGene.cdsStart refGene.cdsEnd refGene.exonCount refGene.exonStarts refGene.exonEnds refLink.product geneName.name refSeqSummary.summary
    NM\_198576 chr1 + 995569 1031415 995619 1030284 36 995569,997647,1010723,1016111,1016522,1016827,1017258,1018541,1018840,1019125,1019411,1019636,1020463,1020661,1021035,1021266,1021462,1021699,1022122,1022629,1022875,1023078,1023314,1024169,1024538,1024868,1025205,1025535,1025729,1026028,1026555,1026755,1027030,1029055,1029750,1030126, 995820,997909,1010771,1016327,1016747,1017052,1017465,1018760,1019035,1019326,1019560,1019742,1020580,1020826,1021179,1021391,1021568,1022038,1022260,1022757,1022990,1023198,1023668,1024362,1024754,1025098,1025340,1025632,1025894,1026140,1026672,1026948,1027118,1029280,1029854,1031415, agrin AGRIN Agrin is a neuronal aggregating factor that induces the aggregation of ...

---


### main

```
public static void main(java.lang.String[] args)
```

---


### processArgs

```
public void processArgs(java.lang.String[] args)
```

:   This method will process each argument and assign new varibles

---


### printDocs

```
public static void printDocs()
```


---


|  |  |  |
| --- | --- | --- |
| **Overview** \| **Package** \| **Class** \| **Use** \| **Tree** \| **Deprecated** \| **Index** \| **Help** | |  |
| **PREV CLASS**   **NEXT CLASS** | **FRAMES**    **NO FRAMES**     **All Classes** |  |
| SUMMARY: NESTED \| FIELD \| CONSTR \| METHOD | DETAIL: FIELD \| CONSTR \| METHOD |  |


---
